# The Effect of the Selective N-methyl-D-aspartate (NMDA) Receptor GluN2B Subunit Antagonist CP-101,606 on Cytochrome P450 2D (CYP2D) Expression and Activity in the Rat Liver and Brain

**DOI:** 10.3390/ijms232213746

**Published:** 2022-11-08

**Authors:** Anna Haduch, Ewa Bromek, Renata Pukło, Joanna Jastrzębska, Przemysław Jan Danek, Władysława Anna Daniel

**Affiliations:** Department of Pharmacokinetics and Drug Metabolism, Maj Institute of Pharmacology, Polish Academy of Sciences, Smętna 12, 31-343 Kraków, Poland

**Keywords:** CP-101,606, CYP2D, activity/expression, liver, brain, chronic treatment

## Abstract

The CYP2D enzymes of the cytochrome P450 superfamily play an important role in psychopharmacology, since they are engaged in the metabolism of psychotropic drugs and endogenous neuroactive substrates, which mediate brain neurotransmission and the therapeutic action of those drugs. The aim of this work was to study the effect of short- and long-term treatment with the selective antagonist of the GluN2B subunit of the NMDA receptor, the compound CP-101,606, which possesses antidepressant properties, on CYP2D expression and activity in the liver and brain of male rats. The presented work shows time-, organ- and brain-structure-dependent effects of 5-day and 3-week treatment with CP-101,606 on CYP2D. Five-day treatment with CP-101,606 increased the activity and protein level of CYP2D in the hippocampus. That effect was maintained after the 3-week treatment and was accompanied by enhancement in the CYP2D activity/protein level in the cortex and cerebellum. In contrast, a 3-week treatment with CP-101,606 diminished the CYP2D activity/protein level in the hypothalamus and striatum. In the liver, CP-101,606 decreased CYP2D activity, but not the protein or mRNA level, after 5-day or 3-week treatment. When added in vitro to liver microsomes, CP-101,606 diminished the CYP2D activity during prolonged incubation. While in the brain, the observed decrease in the CYP2D activity after short- and long-term treatment with CP-101,606 seems to be a consequence of the drug effect on enzyme regulation. In the liver, the direct inhibitory effect of reactive metabolites formed from CP-101,606 on the CYP2D activity may be considered. Since CYP2Ds are engaged in the metabolism of endogenous neuroactive substances, it can be assumed that apart from antagonizing the NMDA receptor, CP-101,606 may modify its own pharmacological effect by affecting brain cytochrome P450. On the other hand, an inhibition of the activity of liver CYP2D may slow down the metabolism of co-administered substrates and lead to pharmacokinetic drug–drug interactions.

## 1. Introduction

The CYP2D enzymes of the cytochrome P450 superfamily play an important role in psychopharmacology, since they are engaged in the metabolism of psychotropic drugs and endogenous neuroactive substrates, which mediate brain neurotransmission and the therapeutic action of those drugs [1,2,3]. Therefore, changes in the activity of CYP2D enzymes, such as human CYP2D6 (liver and brain enzyme) or rat CYP2D1, CYP2D2 (liver-specific) and CYP2D4 (brain-specific), are of particular interest as they can affect drug response via both pharmacokinetic and neurochemical mechanisms, the latter of which include the synthesis of monoaminergic neurotransmitters dopamine [4,5,6] and serotonin [7,8,9], and the metabolism of neurosteroids [10,11,12].

The mechanisms of the regulation of CYP2D enzymes are still not fully recognized, but the influence of age, stress, steroid hormones and neuroactive drugs has been observed [13,14,15,16,17,18]. At a molecular level, the engagement of hepatocyte nuclear factor HNFα, CCAAT/enhancer-binding protein C/EBPα transcription factor, the farnesoid X receptor-activated transcriptional repressor SHP, PPAR nuclear receptors (PPARγ-activating and PPARα-suppressing) and miRNAs in the regulation of *CYP2D6* expression was observed (reviewed by [19,20]). It has also been shown that PPARs are controlled by growth hormone [21], while growth hormone is under the influence of sex steroids [22]. Moreover, sex hormones can regulate brain *CYP2D* genes via miRNAs [23].

The regulation of cytochrome P450 in the brain proceeds differently than in the liver and is brain-region-dependent [3,24]. The brain consists of various structures that contain different cell types, innervations and receptors. It seems, therefore, that differences in intracellular neurotransmitter signaling, availability of endogenous and exogenous active substances dependent on the blood–brain barrier (BBB) permeability and levels of transcription factors between neural and hepatic cells lead to differentiated expression of cytochrome P450 and its susceptibility to regulation at transcriptional or post-transcriptional level in the brain (or brain structures) and the liver. Brain CYP2Ds are usually regulated by xenobiotics at the post-transcriptional level in contrast to the liver enzyme, as shown for some psychotropic drugs and nicotine [3,25,26,27,28].

Human CYP2D6 polymorphism has been implicated in the inter-individual variability of therapeutic responses, personality traits, cognitive processes, learning and memory [29,30,31,32,33,34]. This kind of relationship between CYP2D activity and behavioral responses was also observed in rodents [21,35,36]. Recent studies have shown that prolonged administration of some antidepressant and neuroleptic drugs affects CYP2D activity in brain structures involved in depression and schizophrenia (respectively), and influences the therapeutic action or side effects of those drugs (reviewed by [3]). Since CYP2Ds are engaged in the metabolism of endogenous neuroactive substances in the brain, changes in their activity may modify the pharmacological action of psychotropics, as recently suggested for the investigated antidepressants (e.g., escitalopram, venlafaxine) and atypical neuroleptics (e.g., asenapine, iloperidone and lurasidone) [15,27,37,38,39]. The abovementioned three atypical neuroleptics evoked similar pattern of changes in the regional CYP2D activity in the brain, which was different from that of antidepressants, and suggested the involvement of pharmacological (receptor) mechanisms.

Recent studies indicate that selective antagonists of the GluN2B subunit of the N-methyl-d-aspartate (NMDA) receptor may be useful for the therapy of major depressive disorders. However, an involvement of the glutamatergic system in the physiological regulation of cytochrome P450 enzymes has not been well-examined. Our recent results with the selective antagonist of the GluN2B subunit of the NMDA receptor, the compound CP-101,606, which possesses antidepressant properties [40,41,42,43], imply engagement of the NMDA receptor in the neuroendocrine regulation of CYP1A, CYP2C and CYP3A enzymes in the liver and potential involvement of drugs acting on NMDA receptors in metabolic drug–drug interactions [44]. However, the effect of CP-101,606 on the CYP2D enzyme has not been investigated. Therefore, the aim of this work was to study the effect of prolonged treatment with the compound CP-101,606 on CYP2D expression and activity in the rat liver and brain. We would like to find out whether the action of this potential antidepressant drug on the CYP2D enzyme may be important for its pharmacological effect in the brain and for metabolic drug–drug interactions in the liver.

## 2. Results

### 2.1. The Effect of CP-101,606 on the CYP2D Protein Level and Activity in Rat Brain Microsomes after 5-Day and 3-Week Treatment

**Figure 1:**
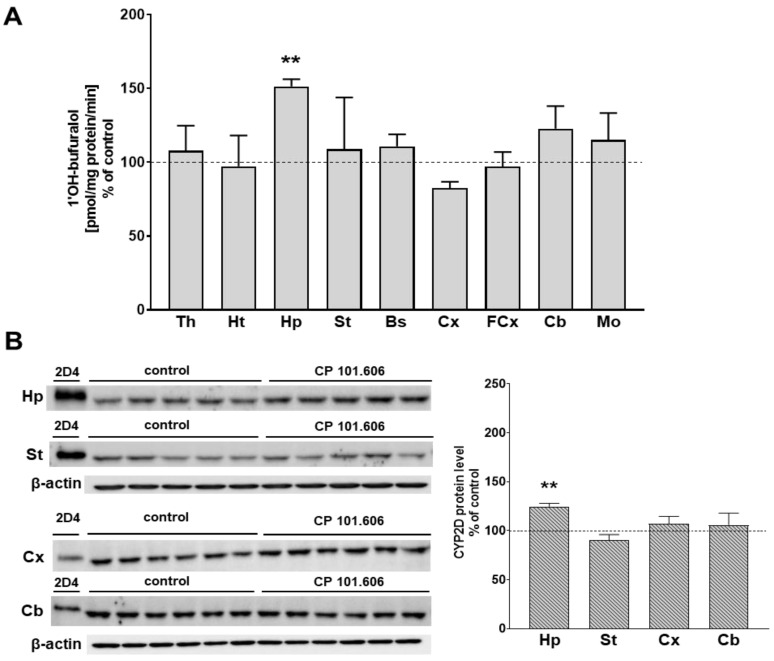
The influence of 5-day treatment with CP-101,606 on the CYP2D activity (**A**) and the protein level (**B**) in microsomes from the selected brain regions. All values are the mean ± S.E.M. of 4–5 samples (each sample consisted of 2 pooled brain structures from 2 rats) for most of the studied cerebral structures or 6 samples (from 6 rats) for the cortex and the cerebellum. The mean values of the control CYP2D activity are as follows (pmol of 1′-hydroxybufuralol/mg protein/min): 0.024 ± 0.003 (Th); 0.024 ± 0.005 (Ht), 0.027 ± 0.004 (Hp); 0.083 ± 0.018 (St); 0.037 ± 0.006 (Bs); 0.039 ± 0.004 (Cx); 0.019 ± 0.002 (FCx); 0.068 ± 0.011 (Cb); and 0.041 ± 0.011 (Mo). The representative CYP2D protein bands in Western blot analysis are shown. A total of 10 μg of microsomal protein was subjected to Western blot analysis. cDNA-expressed CYP2D4 (Baktosomes) was used as a positive control. The significance of results was calculated using Student’s *t*-test. Statistical significance is shown as ** *p* < 0.01 vs. control group. Th—the thalamus, Ht—the hypothalamus, Hp—the hippocampus, St—the striatum, Bs—the brain stem, Cx—the cortex, FCx—the frontal cortex, Cb—the cerebellum, Mo—the medulla oblongata.

**Figure 2:**
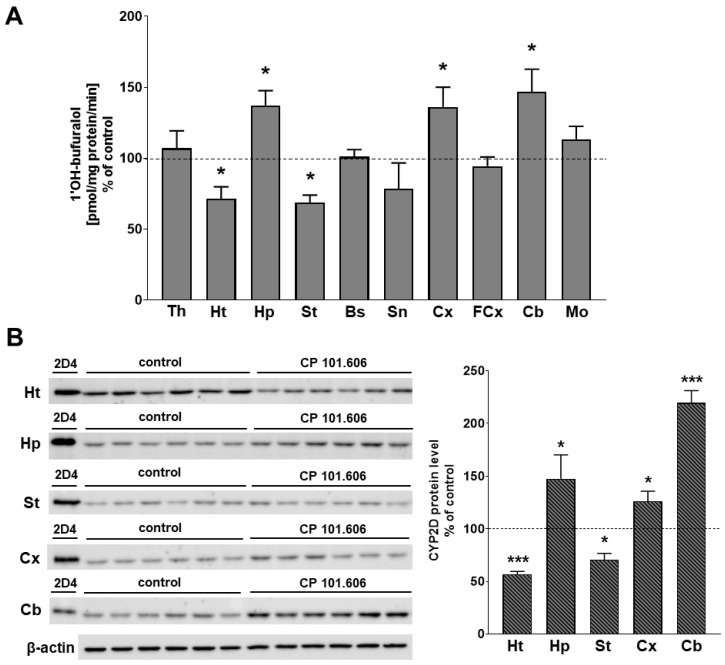
The influence of 3-week treatment with CP-101,606 on the CYP2D activity (**A**) and protein level (**B**) in microsomes from the selected brain regions. All values are the mean ± S.E.M. of 5–7 samples (each sample consisted of 2 pooled brain structures from 2 rats) for most of the studied cerebral structures or 4–5 samples for the substantia nigra (each sample consisted of 3 pooled brain structures from 3 rats) or 6 samples (from 6 rats) for the cortex and the cerebellum. The mean values of the control CYP2D activity are as follows (pmol of 1′-hydroxybufuralol/mg protein/min): 0.035 ± 0.003 (Th); 0.072 ± 0.010 (Ht); 0.031 ± 0.003 (Hp); 0.065 ± 0.007 (St); 0.033 ± 0.005 (Bs); 0.126 ± 0.017 (Sn); 0.023 ± 0.002 (Cx); 0.010 ± 0.001 (FCx); 0.047 ± 0.007 (Cb); and 0.025 ± 0.003 (Mo). The representative CYP2D protein bands in Western blot analysis are shown. A total of 10 μg of microsomal protein was subjected to Western blot analysis. cDNA-expressed CYP2D4 (Bactosome) was used as a positive control. The significance of results was calculated using Student’s *t*-test. Statistical significance is shown as * *p* < 0.05; *** *p* < 0.001 vs. control group. Th—the thalamus, Ht—the hypothalamus, Hp—the hippocampus, St—the striatum, Bs—the brain stem, Sn—the substantia nigra, Cx—the cortex, FCx—the frontal cortex, Cb—the cerebellum, Mo—the medulla oblongata.

The activity of CYP2D, measured as the rate of bufuralol 1′-hydroxylation, and the protein level of the enzyme were not significantly changed in most of the investigated brain structures (the thalamus, hypothalamus, striatum, brain stem, cortex, frontal cortex, cerebellum and medulla oblongata) after 5-day treatment with CP-101,606. One exception was the hippocampus, in which a short-term treatment with CP-101,606 significantly increased the activity of CYP2D up to 151% of the control value and the level of the enzyme protein up to 125% (Figure 1 and Figure S1). The considerably increased activity and protein level of CYP2D in the hippocampus were maintained after 3-week administration of CP-101,606 and amounted to 137% and 148% of the controls, respectively (Figure 2 and Figure S2). Furthermore, the long-term treatment with CP-101,606 enhanced the CYP2D activity in the cortex and cerebellum (up to 136% and 147%, respectively). The elevated activity of CYP2D positively correlated with an increase in the level of the enzyme protein (up to 126% and 219%, respectively). In contrast, a 3-week treatment with CP-101,606 significantly diminished the CYP2D activity in the hypothalamus (down to 72%) and in the striatum (down to 69% of the control value). The observed decreases in the enzyme activity were accompanied by a reduction in the enzyme protein level (down to 57% and 71% of the control, respectively) (Figure 2 and Figure S2). No statistically significant changes in CYP2D activity were observed in the other examined brain structures (the thalamus, brain stem, frontal cortex and medulla oblongata).

### 2.2. The Effect of CP-101,606 on the CYP2D Expression and Activity in Rat Liver Microsomes after 5-Day and 3-Week Treatment

**Figure 3:**
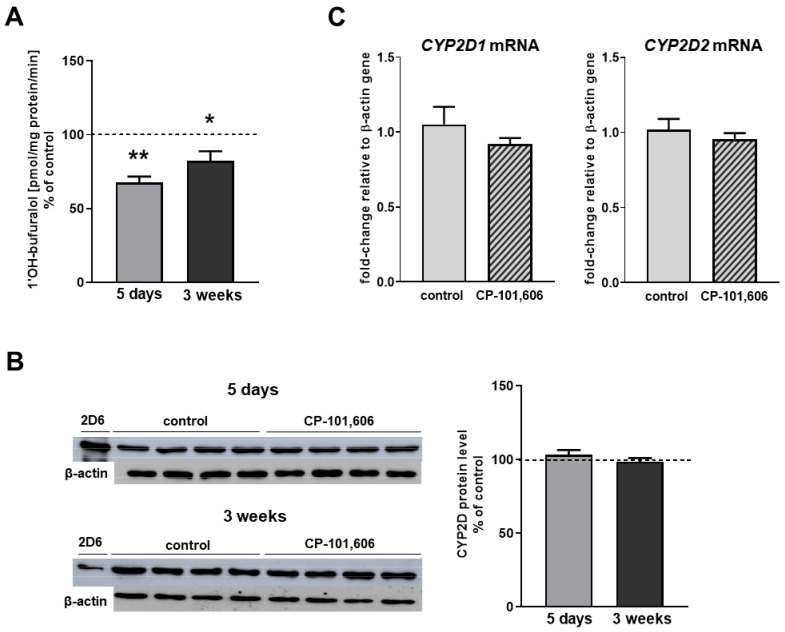
The influence of 5-day and 3-week treatment with CP-101,606 on the CYP2D activity (**A**), the protein level (**B**) and the mRNA levels of *CYP2D1* and *CYP2D2* genes in the liver microsomes (**C**). All values are the mean ± S.E.M. of 9–12 samples. The mean values of the control CYP2D activity are (pmol of 1′-hydroxybufuralol/mg protein/min): 8.827 ± 0.696 (5-day treatment), 11.488 ± 0.549 (3-week treatment). The representative CYP2D protein bands in Western blot analysis are shown. A total of 10 μg of microsomal protein was subjected to Western blot analysis. cDNA-expressed CYP2D6 (Supersome) was used as a positive control. The results are expressed as the fold change in relation to the *ACTB* housekeeping gene. All the values are the means fold change (±S.E.M.) calculated by the comparative delta-delta Ct method (2^−∆∆Ct^) for the control (*n* = 9) and CP-101,606-treated (*n* = 9) groups. The significance of results was calculated using Student’s *t*-test. Statistical significance is shown as * *p* < 0.05; ** *p* < 0.01 vs. control group.

The activity of CYP2D declined to 68% of the control after 5-day administration of CP-101,606. The enzyme activity remained at 82% of that of the control after 3-week treatment with the studied compound (Figure 3). The protein levels of CYP2D were unchanged compared to the control after both 5-day and 3-week treatment with CP-101,606 (Figure 3B and Figure S3). In order to investigate the molecular mechanisms of the observed changes in the CYP2D activity, the mRNA levels of the CYP2D1 and CYP2D2 genes, encoding the major CYP2D enzymes in the liver, were measured. However, CP-101,606 did not produce any significant changes in the mRNA levels of the two studied liver CYP2D genes.

### 2.3. The Effect of CP-101,606 Added In Vitro to Liver Microsomes on the Activity of CYP2D

**Figure 4:**
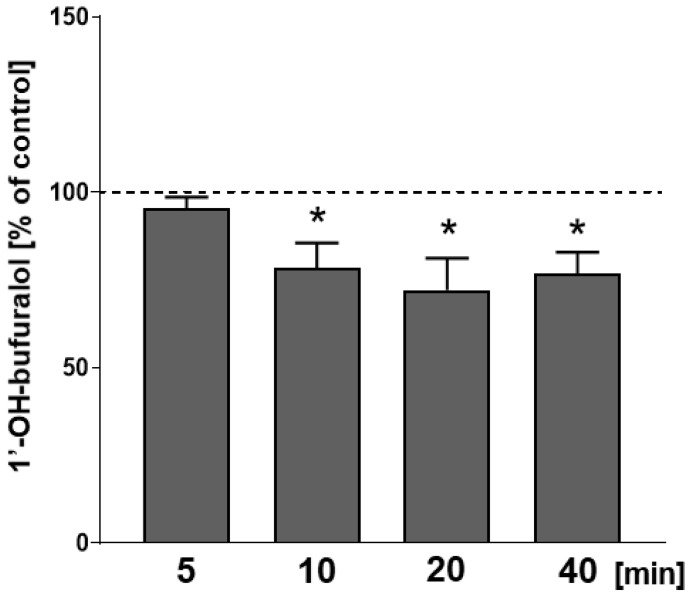
The effect of CP-101.606 added in vitro to pooled liver microsomes from control rats on the activity of CYP2D measured as the rate of bufuralol 1′-hydroxylation at increasing incubation times. All values are the means ± S.E.M. of 5 samples. The significance of results was calculated using Student’s *t*-test. Statistical significance is shown as * *p* < 0.05 vs. control without added CP-101.606. The control values (nmol of 1′-hydroxybufuralol/mg of protein/min) are as follows: 106.10 ± 1.35; 146.24 ± 6.29; 74.55 ± 5.50; 56.55 ± 1.96 (for 5, 10, 20 and 40 min of incubation time, respectively).

In order to further investigate the molecular mechanisms that led to a decrease in CYP2D activity in the liver after the administration of CP-101,606 in vivo, an additional experiment was carried out exclusively in vitro. The studied selective NMDA receptor GluN2B subunit antagonist was added in vitro to liver microsomes of control animals at a concentration of 1 μM. CP-101,606 significantly inhibited the CYP2D activity during prolonged incubation. The compound decreased the rate of bufuralol 1′-hydroxylation by ca. 22% of the control value after 10 min of incubation and by ca. 23% of the control value after 40 min of incubation (Figure 4).

## 3. Discussion

The presented work shows the effect of 5-day and 3-week treatments with the selective NMDA receptor GluN2B subunit antagonist CP-101,606 on cytochrome P450 2D (CYP2D) in the rat brain and liver. The obtained results show time-, organ- and brain-structure-dependent effects of CP-101,606 on the CYP2D enzyme.

While in the liver, the observed decrease in the CYP2D activity after short- and long-term treatment with CP-101,606 does not seem to be a consequence of the drug effect on enzyme regulation, in the brain, such a possibility may be considered. In the liver, no changes in the protein and mRNA levels of the main hepatic CYP2Ds (CYP2D1 and CYP2D2) were observed after in vivo administration of CP-101,606 for 5 days or 3 weeks. However, a decrease in the enzyme activity was observed in vitro as a result of incubation of liver microsomes with the CYP2D-selective substrate bufuralol in the presence of CP-101,606. Therefore, it suggests an inhibitory effect of reactive metabolites formed from CP-101,606 in the liver during drug biotransformation [45] on the CYP2D present therein.

Instead, in the brain, different time- and region-dependent effects of CP-101,606 on CYP2D activity were observed, which were accompanied by respective changes in the CYP2D protein level. Five-day-treatment with CP-101,606 produced an increase in the CYP2D activity and protein level in the hippocampus only, while 3-week treatment resulted in an enhanced enzyme activity and protein level in the hippocampus, cortex and cerebellum. On the other hand, CP-101,606 diminished the CYP2D activity/protein level in the hypothalamus and striatum, and showed such a tendency in the substantia nigra after 3-week treatment. Thus, the above results show an influence of CP-101,606 on the regulation of brain CYP2D (i.e., CYP2D4, the main rat brain CYP2D enzyme), which usually proceeds at a post-transcriptional level, as indicated by previous pharmacological investigations concerning antipsychotic drugs or nicotine [25,26,27,46].

It seems possible that the pharmacological mechanism of CP-101,606 action, i.e., its antagonistic activity at the NMDA receptor GluN2B subunit, plays an important role in the observed differentiated, region-dependent regulation of brain CYP2D. It is known that the brain is not a homogenous organ and consists of many anatomic structures composed of different kinds of neuronal cells (and glial cells), interconnected within the same structure and with other structures via different kinds of neurotransmitter pathways and receptors. Changes in receptor-mediated intracellular signaling may affect pharmacological regulation of genes and proteins including cytochrome P450 [20]. The main glutamate pathways include: cortico-cortical, cortico-brainstem, cortico-striatal, hippocampal-striatal, thalamo-striatal and cortico-thalamic routes [47]. The signals sent from one brain structure to another may be transmitted further (also back) via another neurotransmitter pathway. In addition, the distribution of particular mGluN2 subunits of the NMDA receptor within the brain is different, which may also contribute to the differentiated action of CP-101,606 within the brain [48]. Thus, the intraneuronal and intrastructural signaling evoked by CP-101,606 action at the NMDA receptor may be complex and produce different responses in particular brain regions.

Brain CYP2D has been shown to be induced at a post-transcriptional level, as demonstrated for nicotine, ethanol, clozapine and new atypical neuroleptics [25,26,27,28]. However, detail mechanisms by which drugs affect CYP2D protein levels in the brain have not been recognized so far. Our recent studies indicated similar effects of three atypical neuroleptics, asenapine, iloperidone and lurasidone, acting mainly as antagonists of dopaminergic D2 receptors and serotonergic 5-HT2/6/7 receptors, which affected the CYP2D activity/protein level in different brain regions (e.g., both drugs evoked a decrease in the frontal cortex and cerebellum, but increased it in the striatum) [27,38,39]. The changes produced by these three neuroleptics in brain CYP2D were different from and usually opposite to those found in the present work for the potential antidepressant CP-101,606. The abovementioned examples imply an engagement of brain pharmacological receptors in the differential regulation of cytochrome P450 (CYP2D) in the investigated brain regions. Therefore, further molecular studies are necessary to explain the mechanisms of differentiated regulation of cytochrome P450 (CYP2D) by CP-101,606 and other psychotropic drugs within the brain, considering particular brain structures, neuronal cells and receptors. Studies on neuronal cells and selective receptor ligands may highlight mechanisms of CYP2D protein modulation in particular brain regions and cell types.

Since CYP2Ds are engaged in the metabolism of endogenous neuroactive substances, it can be assumed that apart from antagonizing the NMDA receptor, CP-101,606 may modify its own pharmacological effect by regulating the activity of cytochrome P450 in the brain. The expected decrease in the rate of CYP2D-mediated 21-hydroxylation of neurosteroids (in the hypothalamus and striatum) or acceleration of the synthesis of dopamine and serotonin via alternative pathways (in the hippocampus, cortex or cerebellum) may positively influence the antidepressant action of CP-101,606. On the other hand, the inhibition of the activity of liver CYP2D may slow down the metabolism of co-administered substrates, which are metabolized by this enzyme [49]. Since our previous studies showed that CP-101,606 down-regulates other drug-metabolizing CYP enzymes in the liver, including CYP1A and CYP3A, pharmacokinetic drug–drug interactions should be considered when administering CP-101,606.

## 4. Materials and Methods

### 4.1. Animals

Wistar Han rats from Charles River Laboratories (Sulzfeld, Germany) were used in the study. Males weighing 250–300 g were kept in standard laboratory conditions: temperature 22 ± 2 °C, humidity 55 ± 5% and a 12:12 h light/dark cycle with free access to food and drinking water. All experimental procedures were performed in accordance with the guidelines of the 86/609 EEC Directive and Guide for the Care and Use of Laboratory Animals and with the permission of the Local Bioethics Committee at the Maj Institute of Pharmacology, Polish Academy of Sciences (Kraków, Poland).

### 4.2. Drugs and Chemicals

The selective NMDA receptor GluN2B subunit antagonist CP-101,606 was obtained from Axon Medchem (Groningen, The Netherlands). NADPH, glucose-6-phosphate-dehydrogenase, glucose-6-phosphate and 1′-hydroxybufuralol, the mouse monoclonal anti-β-actin primary antibody, were purchased from Sigma (St. Louis, MO, USA). Bufuralol was synthesized at our institute. All the organic solvents, including HPLC-grade solvents, came from Merck (Darmstadt, Germany). The primary polyclonal anti-rat CYP2D4 antibody (1:1000 dilution) was prepared in a university laboratory at the Osaka City University Medical School (Osaka, Japan), and polyclonal anti-human CYP2D6 antibody (1:2000 dilution) was obtained from Fine Test (Wuhan, China). cDNA-expressed rat CYP2D4 (Bactosome) was donated by Cypex (Dundee, Scotland, UK), and c-DNA expressed CYP2D6 (Supersome) came from Gentest Corp. (Woburn, MA, USA). Goat anti-rabbit peroxidase-conjugated secondary antibodies (1:2000 dilution) were donated by Vector Laboratories (Burlingame, CA, USA), and goat anti-mouse antibodies (1:2000 dilution) were from Jackson ImmunoResearch Laboratories (West Grove, PA, USA). SignalBoostTMImmunoreaction Enhancer Kit for the dilution of the antibodies was delivered by Millipore (Burlington, MA, USA). The chemiluminescence reagents LumiGlo kit came from KPL (Gaithersburg, MD, USA). For RNA isolation, a Total RNA Mini kit purchased from A&A Biotechnology (Gdynia, Poland) was used. A High-Capacity cDNA Reverse Transcription Kit, TaqMan assay and the TaqMan Gene Expression Master Mix were delivered by Life Technologies (Carlsbad, CA, USA).

### 4.3. Animal Procedure and Microsome Preparation

The selective NMDA receptor GluN2B subunit antagonist, named CP-101,606, was administered intraperitoneally in a pharmacologically active dose of 20 mg/kg for 5 days or 3 weeks, simulating short- and long-term treatment [40]. At the end of treatment, the rats were sacrificed by decapitation 2 h after the last dose. Whole livers and brains were removed, and the selected brain structures (the thalamus, hypothalamus, hippocampus, striatum, brain stem, cortex, frontal cortex, substantia nigra, cerebellum and medulla oblongata) were isolated according to the atlas of Paxinos and Watson [50]. All those tissues were immediately frozen in dry ice, and stored at −80 °C until analysis. The differential centrifugation methods described by Hiroi et al. [10] and Daniel et al. [51] were used to prepare liver and brain microsomes. The microsomal suspension was dispensed in portions into Eppendorf vials, which were stored at −80 °C.

### 4.4. Determination of Cytochrome P450 Enzyme Activities in Brain and Liver Microsomes

The activity of the CYP2D was studied by measurement of the rate of a CYP2D-specific metabolic reaction, i.e., bufuralol 1′-hydroxylation [52], in microsomes derived from selected brain structures or livers from control rats or CP101,606-treated animals. Incubations of bufuralol with microsomes from the examined brain structures or liver microsomes were carried out in a system containing the following components: potassium phosphate buffer (2 mM, pH = 7.4), MgCl_2_ (4 mM), NADP (1.6 mM), glucose 6-phosphate (5 mM) and glucose 6-phosphate-dehydrogenase (2.5 U in 0.4 mL), according to the method routinely used in our laboratory. The protein concentrations in the incubated samples with liver microsomes were ca. 0.5 mg of protein/mL, and in samples with brain microsomes concentrations varied depending on the structure of the brain from 1 to 2 rats (mg of protein/mL: ca. 2.71 for the thalamus, 0.33 for the hypothalamus, 1.68 for the hippocampus, 0.52 for the striatum, 1.54 for the brain stem, 3.69 for the cortex, 5.33 for the frontal cortex, 1.44 for the cerebellum and 1.99 for the medulla oblongata) or from 3 rats (ca. 0.1 mg of protein/mL for the substantia nigra). The total level of protein in brain and liver microsomes was measured according to Lowry et al. [53].

Bufuralol was added to the incubation mixture in vitro at a concentration of 5 µM for liver microsomes and 125 µM for brain microsomes (concentrations adjusted according to the differences in non-specific substrate binding and in proportion of CYP2D enzymes between brain and liver) [15,37]. The final incubation volume was 0.4 mL. In the inhibition studies with liver microsomes, CP-101,606 was added to the reaction mixture at a concentration of 1 µM and bufuralol at a 10 µM concentration. The incubation at 37 °C lasted: 60 min for brain microsomes, 10 min for liver microsomes or 5–40 min in the presence of CP-101,606 in control liver microsomes. At the end of the incubation, the reaction was stopped by adding 30 µL of perchloric acid (70%) and placing the samples on ice. After centrifugation (10 min, at 1000× *g*) of the samples, the supernatants were transferred to new Eppendorf vials and stored at −20 °C until further analysis. Concentrations of 1′-hydroxybufuralol produced from bufuralol in brain and liver microsomes were evaluated by a high-performance liquid chromatography method (HPLC) based on Hiroi et al. [10]. The HPLC system (LaChrom, Merck-Hitachi) included the following elements: L-7485 fluorescence detector, an L-7100 pump and D-7000 System Manager and the analytical column Econosphere C18 5 µM, 4.6 × 250 mm (Alltech, Carnforth, England). The conditions for the elution of the assayed compounds were as follows: the mobile phase contained 40% acetonitrile and 0.075% triethylamine (*v*/*v*; pH = 3.0), the flow rate was 1.0 mL min^−1^ from 0.0 to 6.0 min and 1.8 mL min^−1^ from 6.1 to 18.0 min, the column temperature was 50 °C and an excitation wavelength of 252 nm and emission wavelength of 302 nm (emission) were used.

### 4.5. Analysis of CYP2D Proteins in Brain and Liver Microsomes

The CYP2D protein levels in the brain and liver microsomes of control or CP-101,606-treated rats (5-day or 3-week treatment) were estimated by Western immunoblot analysis. Microsomal proteins (5–10 µg of brain and 10 µg of liver microsomes) were separated using SDS polyacrylamide gel electrophoresis and transferred onto nitrocellulose membranes. The proteins were then immunodetected and visualized by chemiluminescence using the method described earlier [37,54].

CYP2D6 is the only human CYP2D enzyme, existing in the liver and brain. In the rat, CYP2D4 is the predominant CYP2D enzyme in the brain, while CYP2D1 and CYP2D2 are the main CYP2D enzymes present in the liver. Therefore, the polyclonal rabbit anti-rat CYP2D4 antibody, which binds to the main rat brain CYP2D enzyme CYP2D4 (and other CYP2Ds), was used as primary antibody for brain microsomes, while the polyclonal rabbit anti-human CYP2D6 antibody, which recognizes all rat CYP2Ds, was used as primary antibody for liver microsomes. Then, horseradish-peroxidase-labeled goat anti-rabbit IgG was used as a secondary antibody. cDNA-expressed CYP2D4 (2.5 µg) and CYP2D6 (1 µg) were applied as standards, respectively. The intensity of the bands on a nitrocellulose membrane was measured with the Luminescent Image Analyzer LAS-1000 and quantified by the Image Reader LAS-1000 and Image Gauge 4.0 programs (Fuji Film, Tokyo, Japan). The data were normalized for protein loading based on the β-actin levels.

### 4.6. Analysis of the Expression of Genes Encoding CYP2D Isoforms in the Liver

Due to the small size of rat brain structures (e.g., ca. 50 mg for the hypothalamus and 140 mg for the hippocampus), the CYP2D mRNA was measured only in the liver. The mRNAs of main liver CYP2D enzymes (CYP2D1 and CYP2D2) were analyzed. Isolation of liver RNA and quantitative real-time PCR (qRT-PCR) were carried out according to the methods described earlier [54]. The frozen liver tissue was homogenized, and total RNA was isolated using a Total RNA Mini kit. The quantity and quality of the isolated RNA were verified using a Synergy/HTX multi-mode reader (BioTek, Winoosk, VT, USA). The isolated RNA samples were stored at −20 °C until use. The first strand of cDNA products was generated (from 1 μg of isolated RNA) using a high-capacity cDNA reverse transcription kit. cDNA synthesis was carried out at 2 °C for 10 min, 37 °C for 120 min, 85 °C for 5 min and then at 4 °C for chilling. The expression of genes encoding the CYP2D enzymes, including *CYP2D1* (Rn01775090_mH) and *CYP2D2* (Rn00562419_m1), and for reference the gene encoding β-actin *ACTB* (Rn00667869_m1), was detected by a real-time polymerase chain reaction (PCR) using TaqMan Gene Expression Master Mix and species-specific TaqMan-type probes and primers (TaqMan Gene Expression Assay, Life Technologies). Real-time PCR runs were performed using the Bio-Rad CFX96 PCR system (Bio-Rad, Hercules, CA, USA). Gene expression was determined using the 2-delta Ct method using *ACTB* expression as a reference, as described previously [54].

### 4.7. Data Analysis

The obtained results are presented as the mean ± S.E.M. Changes in the brain and liver cytochrome P450 enzyme activities and protein and mRNA levels were statistically assessed using a two-tailed Student’s *t*-test. The results were recognized as statistically significant when *p* < 0.05.

## 5. Conclusions

Since CYP2Ds are engaged in the metabolism of endogenous neuroactive substances, it can be assumed that apart from antagonizing the NMDA receptor, CP-101,606 may modify its own pharmacological effect by regulating the activity of cytochrome P450 in the brain. The expected decrease in the rate of CYP2D-mediated 21-hydroxylation of neurosteroids (in the hypothalamus and striatum) or acceleration of the synthesis of dopamine and serotonin via alternative pathways (in the hippocampus, cortex or cerebellum) may positively influence the antidepressant action of CP-101,606. On the other hand, the inhibition of the activity of liver CYP2D may slow down the metabolism of co-administered substrates, which are metabolized by this enzyme [49]. Since our previous studies showed that CP-101,606 down-regulates other drug-metabolizing CYP enzymes in the liver including CYP1A and CYP3A, pharmacokinetic drug–drug interactions should be considered when administering CP-101,606.

## Data Availability

Data are contained within the article.

## Supplementary Materials

The following supporting information can be downloaded at: https://www.mdpi.com/article/10.3390/ijms232213746/s1.
