# Supplementary material for: The Effect of the Selective N-methyl-D-aspartate (NMDA) Receptor GluN2B Subunit Antagonist CP-101,606 on Cytochrome P450 2D (CYP2D) Expression and Activity in the Rat Liver and Brain
Source: Int J Mol Sci. 2022 Nov 8;23(22):13746. doi: 10.3390/ijms232213746 (PMC9691159; doi:10.3390/ijms232213746)
Supplement: Supplementary file 1 [file ijms-23-13746-s001.zip › ijms-2015229-supplementary.pdf]

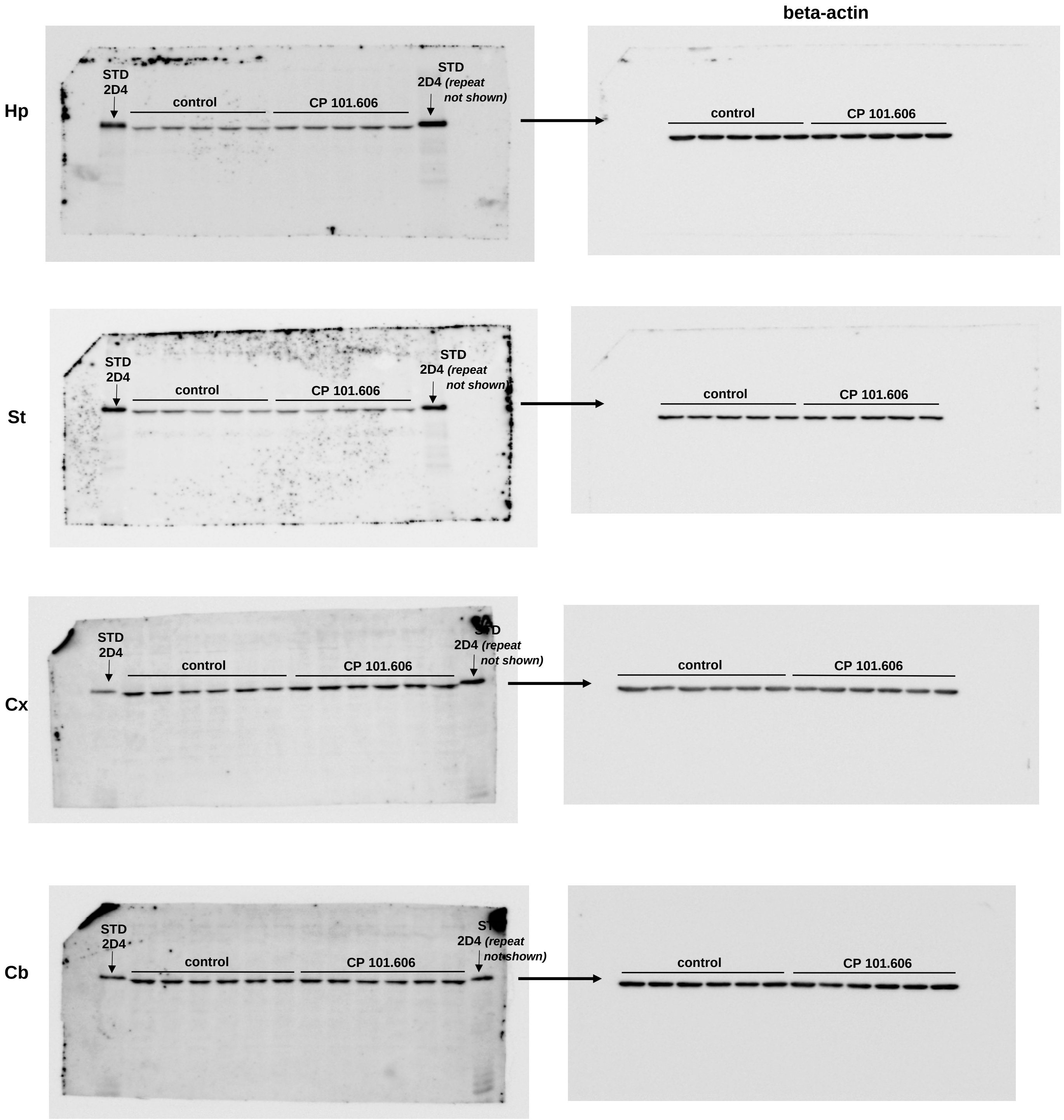

Figure S1. The influence of 5-day treatment with CP-101,606 on the CYP2D protein level in microsomes from the selected brain regions (original membranes to Figure 1B.).

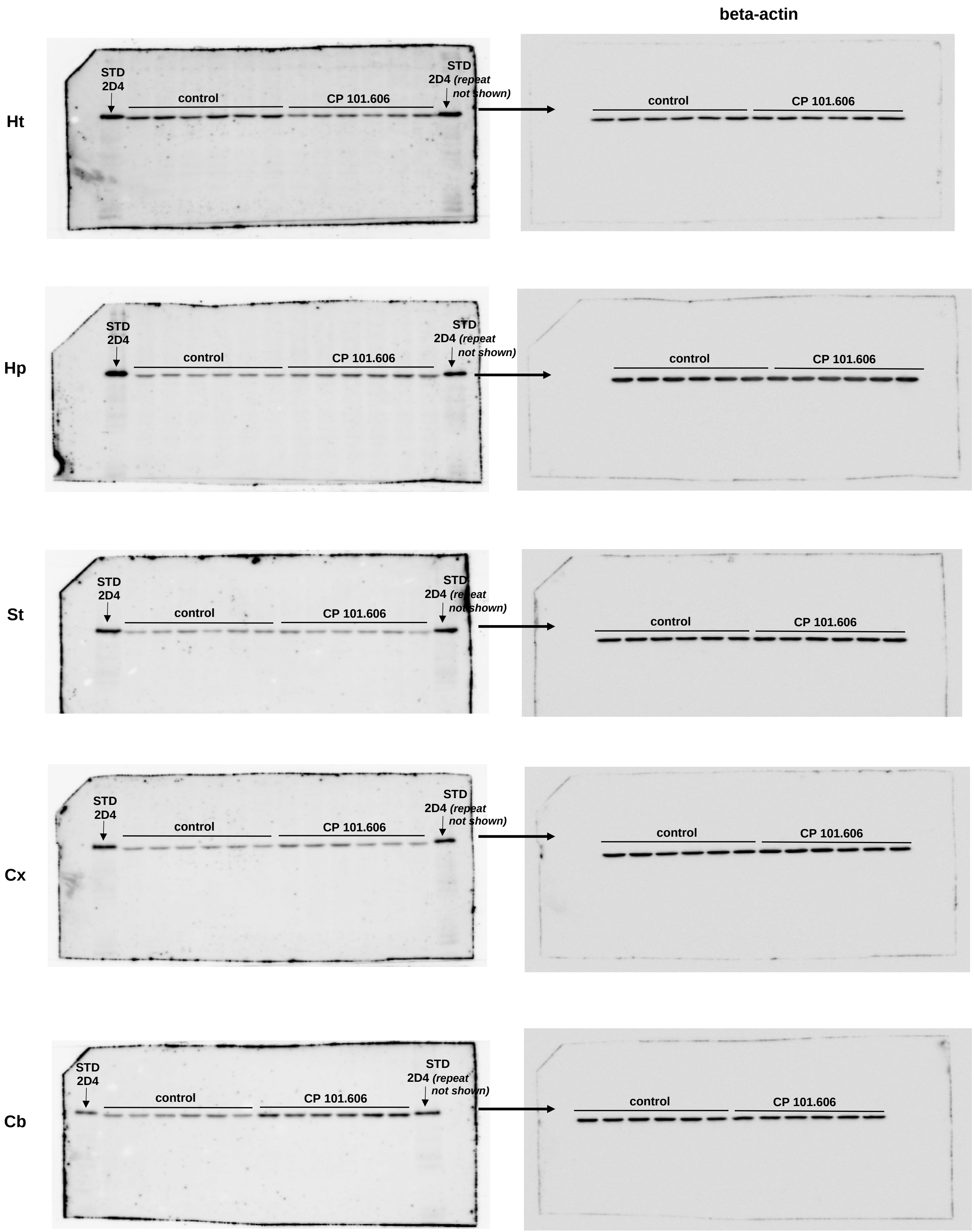

Figure S2. The influence of 3-week treatment with CP-101,606 on the CYP2D protein level in microsomes from the selected brain regions (original membranes to Figure 2B.).

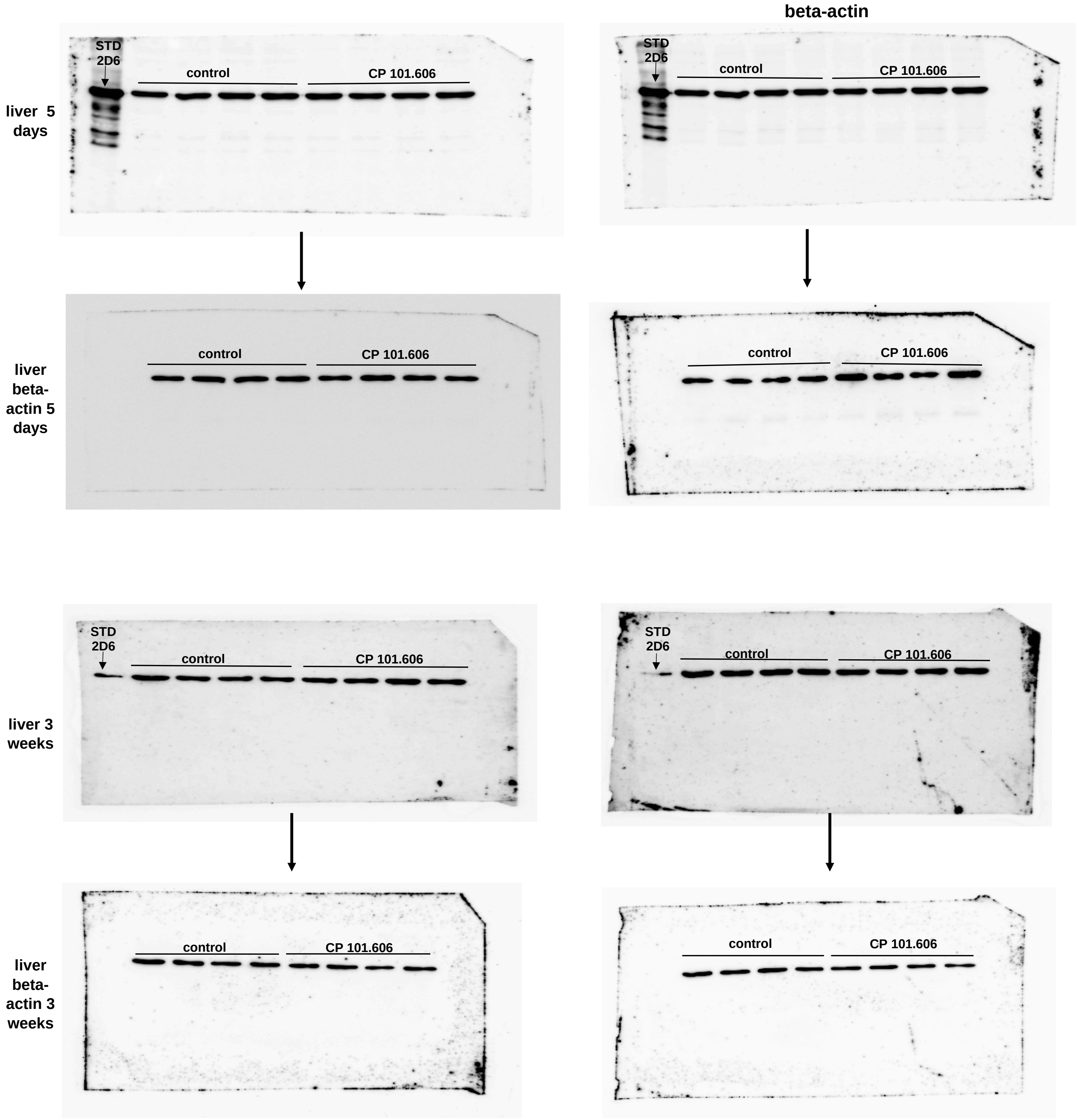

Figure S3. The influence of 5-day and 3-week treatment with CP-101,606 on the CYP2D protein level in the liver microsomes (original membranes to Figure 3B.).
